# Analysis of the Effectiveness of Public Health Measures on COVID-19 Transmission

**DOI:** 10.3390/ijerph20186758

**Published:** 2023-09-14

**Authors:** Thiago Christiano Silva, Leandro Anghinoni, Cassia Pereira das Chagas, Liang Zhao, Benjamin Miranda Tabak

**Affiliations:** 1 Universidade Católica de Brasília, Brasilia 71966-700, Brazil; 2 Department of Computing and Mathematics, Faculty of Philosophy, Sciences, and Literatures in Ribeirão Preto, Universidade de São Paulo, São Paulo 14040-901, Brazil; 3 FGV/EPPG Escola de Políticas Públicas e Governo, Fundação Getúlio Vargas (School of Public Policy and Government, Getulio Vargas Foundation), Brasilia 70830-020, Brazil

**Keywords:** artificial intelligence, COVID-19, network, VAR, SIR

## Abstract

In this study, we investigate the COVID-19 epidemics in Brazilian cities, using early-time approximations of the SIR model in networks and combining the VAR (vector autoregressive) model with machine learning techniques. Different from other works, the underlying network was constructed by inputting real-world data on local COVID-19 cases reported by Brazilian cities into a regularized VAR model. This model estimates directional COVID-19 transmission channels (connections or links between nodes) of each pair of cities (vertices or nodes) using spectral network analysis. Despite the simple epidemiological model, our predictions align well with the real COVID-19 dynamics across Brazilian municipalities, using data only up until May 2020. Given the rising number of infectious people in Brazil—a possible indicator of a second wave—these early-time approximations could be valuable in gauging the magnitude of the next contagion peak. We further examine the effect of public health policies, including social isolation and mask usage, by creating counterfactual scenarios to quantify the human impact of these public health measures in reducing peak COVID-19 cases. We discover that the effectiveness of social isolation and mask usage varies significantly across cities. We hope our study will support the development of future public health measures.

## 1. Introduction

The unprecedented spread of COVID-19 across the globe underscores our world’s significant degree of interconnectedness. In less than half a year, since the initial outbreak in early 2020, the epicenter of the pandemic made a transcontinental journey, commencing in China, traversing to Italy, and eventually reaching the United States. As of 10 March 2023, the Coronavirus Resource Center at Johns Hopkins University (https://coronavirus.jhu.edu/map.html, accessed on 11 March 2023) reported over 676 million cases across more than 188 nations, which approximates to 98% of all countries recognized by the United Nations. Such widespread diffusion can be attributed to factors such as robust international air travel during the pandemic’s early stages, enabling the novel coronavirus to spread globally (refer to a discussion on Argentina’s early response to the COVID-19 pandemic [1]). Despite subsequent international travel restrictions, the virus continued its proliferation within nations for a prolonged duration. The dynamics of this epidemic were dictated not only by local factors within urban areas but also by the perpetual movement of people via roadways, domestic airlines, and maritime routes, promoting the dispersal of the disease [2,3,4]. (Several studies have proposed innovative methodologies to monitor COVID-19 in Europe [5] and to forecast its spread in American metropolitan areas using a spatial SEIR model [6]).

Given this context, we introduce a novel methodology to decipher the COVID-19 transmission network among cities using real-world, chronological data. This versatile model can be adapted to any networked environment and can be implemented in global studies or applied for detailed investigations at the urban level. The proposed network is conceptualized as a weighted directed graph, wherein the vertices symbolize cities, and the links represent potential COVID-19 transmission paths from one city to another. The estimation of these links is derived using a VAR model fitted with high-frequency panel data (observations of multiple cities across time), encompassing city-specific COVID-19 infection counts over time. Our paper makes two significant contributions to the literature. First, we introduce a novel approach that combines the epidemiological model SIR in a networked environment with the estimation of the network using a VAR model. While the use of each technique separately is not novel, their integration with high-frequency data represents a noteworthy contribution. Second, we address a crucial question concerning the dynamics of COVID-19 at a granular level (city) within an emerging country (Brazil).

The primary aim of this study is to construct a predictive model for the initial spread of COVID-19, a period marked by limited data and heightened uncertainty. In emergent pandemic situations such as these, a quandary often arises: the urgent requirement for precise projections to guide strategic decisions clashes with the scarcity of available data and the incomplete understanding of the virus dynamics.

Our predictive model is meticulously designed to suit the context of a pandemic’s initial stages, characterized by high uncertainty surrounding the disease’s potential trajectory and contagiousness. Moreover, we acknowledge that the transmission rate and severity of the disease may substantially differ across diverse populations and regions due to a multitude of socioeconomic, demographic, and environmental factors. Thus, our model accommodates location-specific transmission estimates, bolstering its relevance and pragmatic use.

The significance of our research question to society cannot be overstated, as comprehending the potential progression of a pandemic from its inception enables swifter, more effective responses. Superior projections of disease spread empower decision makers to allocate resources more proficiently, optimizing efforts to curtail the disease and mitigate its effects on public health. We are confident that our efforts constitute a substantial advancement in developing effective predictive models for pandemics’ initial stages, contributing to enhanced societal responses to these critical, large-scale challenges.

We utilized data from 5570 municipalities in Brazil to estimate contagion spread among them. Parameters from the SIR (susceptible, infected, recovered) model, coupled with an autoregressive vector (VAR), were employed to construct the contagion network across municipalities. Estimating a model involving such a number of municipalities within the context of COVID-19 poses a challenge during the pandemic’s early stage. To circumvent this issue, we employed a regularized VAR with elastic net.

An intriguing characteristic of the early-time dynamics in a susceptible–infected–recovered (SIR) model is its capacity to derive the model’s transmission rate β. Given the transmission rate β and the recovery rate γ for infected individuals, the model can be fully characterized [7], including late-time dynamics and peak infection periods. The recovery rate γ can be inferred from the timeline spanning the onset of symptoms to the resolution of the case.

Numerous ongoing studies provide estimates for the recovery rate. For instance, ref. [8] postulates that the infection duration fluctuates between 15 and 20 days. Data derived from the Wuhan outbreak present an onset-to-death duration of 17.8 days and an onset-to-recovery duration of 24.7 days [9]. However, these findings might be skewed towards higher values due to the overwhelmed healthcare system in Wuhan during the outbreak’s initial days and the under-reporting of mild cases’ outcomes. The WHO suggests a recovery duration of 14 days for mild cases and 21–42 days for severe cases. For those who succumb to the disease, the time from onset to outcome varies between 14 and 56 days [10]. As mild cases constitute the majority, we designate γ as 14 days in this study. Subsequently, the model can be fully characterized [7], encompassing late-time dynamics and peak infection periods.

It is pertinent to highlight that the rate γ bifurcates into two parts: the time from onset to death and the time from onset to recovery. These durations may vary among different countries due to their correlation with demographics, healthcare infrastructure, and treatment availability. Nevertheless, the time from onset to recovery remains uninfluenced by the system’s topological structure. Consequently, we adopt an average value of 14 days across all scenarios in our study [10].

We stress that several countries initially underestimated the severity and potential extent of the COVID-19 pandemic, resulting in a constrained initial allocation of containment resources. As healthcare systems started nearing capacity, governments enforced more rigorous measures, such as mandatory social isolation and lockdowns. In this context, we chose to implement an epidemiological model specifically tailored for the early stages of a pandemic. This model is especially valuable due to the scarcity of data at an outbreak’s onset. Nonetheless, it facilitates the prediction of potential disease dispersion. It is a short-term model that leverages linearization and allows parameter identification. It is unsuitable for the later stages of the pandemic, as analyzing these phases involves considering subsequent waves of the disease, where the recovery rate and vaccination coverage turn into relevant variables—data unattainable during a pandemic’s initial phase. We underline that this is a crucial forecasting exercise that can be utilized at the commencement of future pandemics.

In early-time dynamics, the effective transmission rate β of an *isolated SIR* and a *networked SIR* model is distinguished by the spectrum of the estimated COVID-19 transmission network. Without considering the network environment, we effectively presume the existence of a singular, large city composed of all cities in the model. Here, susceptibility to infection depends on the total number of infections across all cities. The introduction of multiple cities effectively curtails this susceptibility by stipulating that the likelihood of being infected is higher within cities than between them. The network spectrum aligns with the largest eigenvalue of the network adjacency matrix. If the isolated SIR model has a transmission rate of β, then the networked SIR model exhibits an effective transmission rate of $\beta_{\mathrm{eff}}=\lambda_{\max}\beta$, where $\lambda_{\max}$ is the largest eigenvalue of the network [7]. The network spectrum encapsulates the entire graph structure in terms of its potential to disperse and amplify intercity contagion at an early stage.

To derive directional COVID-19 transmissions for every pair of cities in the network from the data, we deploy a vector autoregressive (VAR) model. Since its inception in the seminal paper [11], the VAR model has significantly contributed to various economic and financial domains. Nevertheless, its utilization in epidemiology and applied machine learning is a novel concept. Our custom-designed VAR model accommodates the temporal sequence of disease dissemination. We posit that each city-specific infection count depends not just on its historical values, but also on those of every other city. The links in our network essentially represent the weights of past values of city *j* influencing the current infection count of city *i*. These links are estimated by fitting the entire network structure to temporal, city-specific infection count data. We address overfitting concerns by implementing an elastic net regularization scheme during training and employing one-step-ahead rolling validation methodologies, drawing from the machine learning literature.

We utilize a regularized VAR with an elastic net to examine the cross-correlation among municipalities. Given these municipalities’ proximity and the frequent movement of people between them, it was pivotal to model contagion beyond a singular municipality and consider potential contagion from one municipality to another at the onset of a pandemic. It is crucial to emphasize that our focus is on the initial stage. In the medium to long term, exploring each city’s growth and understanding the timeline for the first cases would offer invaluable insights for intercity comparisons. Distinct from other studies, we do not consider each location in isolation but integrate cross-correlation into our analysis. Moreover, we conducted an examination of the contagion network topology among the municipalities.

This paper also scrutinizes the effectiveness of health policy measures enacted by the Brazilian government to mitigate COVID-19 spread. Several states adopted social isolation and quarantine measures at varying timescales. Subsequently, the Brazilian Health Ministry recommended mask usage at the federal level. Political disagreements regarding the efficacy of quarantine measures between federal and state governments were apparent. Our analysis enabled us to identify the principal COVID-19 spreaders at the beginning and peak of the pandemic using initial phase data. Finally, we conducted a counterfactual analysis using graph theory, evaluating the effectiveness of the measures enacted to combat COVID-19.

## 2. Related Background and Literature

This section presents relevant background on SIR models and epidemic spreading in networks and the related literature about our work.

### 2.1. Relevant Background: The Early-Time Dynamic of SIR Models in Networks

This section presents relevant background on the susceptible–infectious–recovered (SIR) model in networks. We refer the reader to [7] for a comprehensive analysis of epidemiological models and to [12] for the seminal paper on the original SIR model. Since we focus on the early-time dynamics of the SIR models, we can assume that the numbers of births and deaths are much smaller than the population, in a way that the closed population hypothesis holds.

Define as $s_{i}\left(t\right)$, $x_{i}\left(t\right)$, and $r_{i}\left(t\right)$ the share of susceptible, infectious, and recovery persons of the city *i* relative to the local population at time *t*. In a closed population, the SIR model in networks is governed by the following differential equations:

(1) $$\begin{array}{cc} s_{i}\left(t+1\right) & =-\beta \cdot s_{i}\left(t\right)\cdot \sum_{j\in V}A_{ij}x_{j}\left(t\right) \end{array}$$

(2) $$\begin{array}{cc} x_{i}\left(t+1\right) & =\beta \cdot s_{i}\left(t\right)\cdot \sum_{j\in V}A_{ij}x_{j}\left(t\right)-\gamma \cdot x_{i}\left(t\right) \end{array}$$

(3) $$\begin{array}{cc} r_{i}\left(t+1\right) & =\gamma \cdot x_{i}\left(t\right) \end{array}$$

(4) $$\begin{array}{cc} 1 & =s_{i}\left(t\right)+x_{i}\left(t\right)+r_{i}\left(t\right) \end{array}$$

∀i∈V and t≥0. We can substitute (4) into (2), yielding

(5) $$\begin{array}{cc} x_{i}\left(t+1\right) & =\beta \cdot \left(1-x_{i}\left(t\right)-r_{i}\left(t\right)\right)\cdot \sum_{j\in V}A_{ij}x_{j}\left(t\right)-\gamma \cdot x_{i}\left(t\right) \end{array}$$

In early times, we can assume that $x_{i}\left(t\right)\ll 1$ and $r_{i}\left(t\right)\approx 0$, ∀i∈V. Therefore, we can ignore second-order $x_{i}\left(t\right)$ terms and effectively set $r_{i}\left(t\right)$ to 0. With these modifications, Equation (5) becomes

(6) $$\begin{array}{cc} x_{i}\left(t+1\right) & =\beta \cdot \sum_{j\in V}A_{ij}x_{j}\left(t\right)-\gamma \cdot x_{i}\left(t\right)\\ \; & =\beta \cdot \sum_{j\in V}\left(A_{ij}-\frac{\gamma }{\beta }\delta_{ij}\right)x_{j}\left(t\right),\\ \; & =\beta \left(A-\left(\frac{\gamma }{\beta }\right)I\right)x\left(t\right)\\ \; & =\beta Mx(t) \end{array}$$

in which *I* is the identity matrix, $M=A-\left(\frac{\gamma }{\beta }\right)I$ is the adjacency matrix *A* with a homogeneous perturbation of $\frac{\gamma }{\beta }$ in the main diagonal, and $\delta_{ij}=1$ if i=j, and $\delta_{ij}=0$ otherwise. Equation (6) is a standard differential linear system whose solution can be written in terms of the eigenvector basis of the adjacency matrix *A*:

(7) $$\begin{array}{c} x_{i}\left(t\right)=\sum_{k=1}^{V}a_{i,k}\left(0\right)e^{\left(\lambda_{k}\beta -\gamma \right)t}v_{i,k}, \end{array}$$

in which $A\cdot v_{k}=\lambda_{k}v_{k}$ holds ∀k∈{1,…,V}. The term $\lambda_{k}$ is the *k*-th eigenvalue of *A*, and $v_{i,k}$ is the *i*-th entry of the eigenvector associated with the *k*-th eigenvalue. The parameter $a_{i,k}\left(0\right)$ in (7) is a scaling constant that depends on the initial condition of the city *i*.

In early times, the growth rate of Equation (7) is governed by the exponent term with the largest eigenvalue $\lambda_{1}=\lambda_{\max}$ of matrix *A*, which is a well-known measure from a spectral-graph-theory-denominated graph spectrum [13]. Therefore,

(8) $$\begin{array}{c} x_{i}\left(t\right)\approx v_{i,1}e^{\left(\lambda_{\max}\beta -\gamma \right)t}, \end{array}$$

i.e., the growth rate is $\lambda_{\max}\beta -\gamma$ and the probability of contagion is proportional to the eigenvector associated with the largest eigenvalue $\lambda_{\max}$, $v_{1}$, which corresponds to the eigenvector centrality measure of the graph, according to the spectral graph theory [13].

### 2.2. Relevant Background: To Prevent Epidemic Spreading in Networks

In the study of epidemic prevention in networks, there are two main strategies [14]. The first is the use of efficient immunization protocols, and the second involves identifying the relevant spreaders and activation mechanisms. Immunization protocols are methods for identifying nodes that should be immunized, taking into account the network structure. Immunized nodes and all incident links can be removed from the epidemic network, protecting the immunized individuals and reducing the epidemic threshold, thereby preventing the disease outbreak.

The random immunization protocol is the simplest among various immunization strategies, where a fraction of randomly selected nodes are made immune. However, in this case, the immunization threshold tends to be 1 in heterogeneous networks, indicating that almost the entirety of the network must be immunized to suppress the disease [15]. Target immunization protocol considers special nodes to be immunized. It has been shown that the immunization threshold can be exponentially small over a wide range of the spreading rate if it considers the immunization of a fraction of nodes with the highest degree [15,16]. Other approaches consider the critical nodes and the entire prevalence curve (the so-called viral conductance) [17,18].

Even though immunization is a fundamental strategy in the study of epidemics, the scientific community has also devoted a lot of attention to identifying which nodes, links, and local structures are most influential or effective in the spreading process [19,20,21,22,23,24,25,26]. These findings aim to understand network measures on nodes and links, such as degree, betweenness, K-core index, closeness, and link property in the dynamics of spreading. However, the strategies mentioned require the discovery of a vaccine or at least partial knowledge of the epidemic network under consideration. With the mass and rapid spread of COVID-19, neither of them is a practical method to prevent the outbreak. Therefore, global intervention methods, such as social distancing and even lockdowns, have proven to be efficient. For this reason, we study the effectiveness of public intervention methods [27,28,29].

Our results provide strong evidence of the effectiveness of public health measures, such as quarantine and the use of masks, to reduce the infection rate even without detailed information about the highly dynamic population network. A reduction in the incidence of COVID-19 due to handwashing, mask use, and distancing was identified [2]. Due to the heterogeneity of the studies, it was impossible to conduct a meta-analysis of the effects of quarantine and isolation, universal lockdowns, and the closure of borders, schools, and workplaces.

The use of a meta-analysis to conclude that public health interventions and nonpharmaceutical measures successfully reduced COVID-19 transmission was effective [30]. Included studies demonstrated that travel restrictions, border measures, quarantine of travelers arriving from affected regions, city lockdowns, mass gathering restrictions, isolation and quarantine of known cases and close connections, social distancing measures, mandatory mask use, contact tracing, testing, school closures, and the use of personal protective equipment by healthcare workers were effective in preventing the spread of COVID-19.

On the other hand, it was found that closing all academic institutions, restricting gatherings to 10 people or fewer, and shutting down face-to-face businesses significantly reduced transmission. The additional impact of stay-at-home orders was negligible [31].

It was shown that the effectiveness of nonpharmaceutical interventions (NPIs) to mitigate the spread of SARS-CoV-2 depends on local contexts, such as the timing of the adoption of such measures and socioeconomic, political, and cultural features [32]. Our paper contributes to this literature by including how the cross-correlation between cities also matters for contagion in the early stages of the pandemic.

## 3. Data and Methods

### 3.1. Data

We use daily data on the number of infectious persons per city in Brazil using COVID-19 epidemiological bulletins of 27 state health departments from 25 February 2020 to 8 May 2020. (These data are scattered around a large quantity of state government sites. The bulletins are generally not standardized across different states and not even cities. We use the compiled dataset from Brasil.io for this task). A paneldata set is composed of city-specific COVID-19 infection counts over time. It is a mixture of cross-sectionaldata, in which we observe *n* cities all in a specific time point, and timeseriesdata, in which we observe a single individual over time. Each Brazilian state compiles local reports from cities inside their geographical circumscription. We ended up with 60,021 city-time epidemiological bulletins comprising 2754 (out of 5570) cities affected by COVID-19 in Brazil.

Our data are representative because local hospitals are required by law to register any COVID-19 events to the local government, while cities and states must notify the federal government. However, there may be substantial subnotifications due to persons that acquire COVID-19 and recover un-noticed or without hospitalization. (Asymptomatic and mild cases can represent up to 80% of the cases according to China’s reported numbers. These cases tend not to be tested in Brazil).

We also collect city-level population estimates from the Brazilian Institute of Geography and Statistics (IBGE), which is the agency responsible for the official collection of statistical, geographic, cartographic, geodetic, and environmental information in Brazil. We evaluate the share of infectious persons by taking the ratio of COVID-19 cases reported in the local health bulletin and the local population size. The use of shares in our estimation models is important because it is a stationary variable.

We apply a 3-day smoothing filter on the number of infectious persons in each municipality to alleviate concerns with late contamination reports or short-term rectifications by the local health government that could compromise our estimations. In our network construction procedure (see Section 3.2.1), we keep only cities that reported COVID-19 cases in at least 20% of the available time frame. Our results remain qualitatively the same if we do not apply this filtering criterion. In our estimation of the SIR parameters (see Section 3.2.2), we center all time points in relation to the occurrence of the first death in the city.

### 3.2. Methods

Our analysis consists of the following stages:

- Networkconstruction: we construct the COVID-19 network transmission network by fitting the network links to real data.
- *COVID-19 epidemics estimation using the SIR model*: we use the network estimated in Step 1 and simulate the COVID-19 evolution in every city of the network.
- *Effectiveness evaluation of public health policy*: we change the network structure so as to simulate the omission of public health policies and run our epidemics model in Step 2 without government intervention. We estimate the efficiency of public health policies by inspecting the change in the COVID-19 epidemic’s peak.

#### 3.2.1. Network Construction Using Panel Data

Consider the weighted directed graph G=〈V,E〉 in which V is the set of vertices and E is the set of links. There are V=|V| vertices and E=|E| links in the network. In our epidemiological application, vertices can represent cities, states, countries, or any well-defined entity or geographical circumscription (e.g., neighborhood, street, house). We denominate the vertices as cities for simplicity and with no loss of generality. We assume as given the set of cities/vertices V. In contrast, links between cities *i* and *j* connote potential COVID-19 transmission from *i* to *j* and are apriori unknown. In the context of cities, city-to-city contagion could happen for several reasons, such as when infectious persons visit or migrate or even from intercity transportation of supplies covered in surfaces where SARS-CoV-2 is viable for long periods. Therefore, the network G encodes all potential transmission paths between cities through organic or nonorganic media. The goal of this section is to estimate the set of links E, i.e., the intercity COVID-19 transmission channels.

Let $x\left(t\right)=\left[x_{1}\left(t\right),x_{2}\left(t\right),\ldots ,x_{V}\left(t\right)\right]$ denote the vector with shares of infectious persons relative to the local population of every city i∈V in the network at discrete time t≥0. Specifically, we denote as $x_{i}\left(t\right)\in \left[0,1\right]$ the share of infectious persons within city *i* at time *t*. That is, we take the ratio between the number of infectious persons and the city’s local population. When $x_{i}\left(t\right)=1$, all city population is infectious. When $x_{i}\left(t\right)=0$, none is infectious. In-between values represent partial shares of the infectious population. Define the column vector $x_{i}={\left[x_{i}\left(0\right),x_{i}\left(1\right),\ldots ,x_{i}\left(T\right)\right]}^{\prime }$ as the COVID-19 time series evolution in city *i* up to time *T*, in which the superscript ′ is the transpose operator. Since we perform an early-time analysis of the epidemics, *T* is likely not to be large. Let also the matrix $X=[x_{1},x_{2},\ldots ,x_{V}]$, $\dim\left(X\right)=T\times V$, be all the cities’ time series with the shares of infectious persons stacked in columns throughout all period with available data (panel data).

To construct the network, we consider the temporal ordering of the COVID-19 spread across different cities. We attempt to describe the current share of infectious person vector $x_{t}$ with the same vector immediately at the previous time step, i.e., $x_{t-1}$, as follows:

(9) $$\begin{array}{c} x_{t}=\kappa +A\cdot x_{t-1}+\epsilon_{t}, \end{array}$$

∀t∈{0,1,…,T}. The term κ, dim(κ)=V×1, is an intercept column vector; *A*, dim(A)=V×V, is the adjacency matrix encoding the set of links E of the graph; and $\epsilon_{t}∼\left(0,\Sigma_{\epsilon }\right)$ is the unobservable zero mean white noise vector process (serially uncorrelated or independent) with time-invariant covariance matrix $\Sigma_{\epsilon }$. Let $A_{ij}$ be the (*i*,*j*)-entry of *A*, i,j∈V. When $A_{ij}>0$, then city *i* can spill over COVID-19 to city *j*. The larger $A_{ij}$ is the stronger such contagion. Then, the set of links is given by $E=\{i,j\in V:A_{ij}>0\}$.

The terms κ, *A* in Equation (9) are unknown and are estimated using a fitting process to the observed data *X*. Equation (9) describes a VAR(1) model. The companion matrix must have roots inside the complex unit circle to ensure the system is stable. To guarantee such property, our variables $x_{i},i\in V$, must be stationary. Since they are lower- and upper-bounded—i.e., $x_{i}\in \left[0,1\right]$—, they are stationary by construction. Specifically, we minimize the following regularized loss function *L* [33] using the coordinate descent algorithm [34]:

(10) $$\begin{array}{cc} L & =\min_{\kappa ,A}\sum_{t=0}^{T}{\epsilon_{t}}_{F}^{2}+\mathrm{Regularization}\left(A\right)\\ \; & =\min_{\kappa ,A}\sum_{t=0}^{T}{y_{t}-\left(\kappa +Ax_{t-1}\right)}_{F}^{2}+\lambda \left(\alpha A_{1}+\left(1-\alpha \right)A_{2}\right), \end{array}$$

in which λ≥0 is the *elastic net* regularization term and α∈[0,1] is the tradeoff parameter between lasso ($L_{1}$ norm) and ridge ($L_{2}$ norm) regularizations. We notate $._{F}$, $._{1}$, $._{2}$ as the Frobenius, $L_{1}$, and $L_{2}$ norms, respectively. Larger values of λ encourage sparser networks. The first term represents the minimization of the error term $\epsilon_{t},\forall t\in 0,1,\ldots ,T$, and ensures that the estimated adjacency matrix *A* better reflects the COVID-19 transmission dynamics over time. The second term is a regularization term over the adjacency matrix *A* introduced to prevent overfitting and ensure that the estimation is numerically tractable. We do not regularize the intercept vector κ because it conceptually adapts to the city-specific average values of our data.

There is an empirical challenge in fitting the adjacency matrix *A* to the panel data *X* when we are dealing with large-scale networks in which the number of cities *V* largely surpasses the number of available time points *T*, i.e., when V≫T. Such a problem is aggravated when we only have early-time information about the disease; i.e., *T* is small. In this case, we would incur overparametrization and overfitting is a concern. The regularization term in (10) mitigates such concern. We opt for an *elastic net* regularization scheme because it is a robust regularization that combines positive features of lasso and ridge regularizations [34].

Due to the temporal dependency of the panel data, the usual *k*-fold cross-validation is not well suited for our model selection procedure. Following [33], we optimize the penalty parameters λ and α in (10) using an *h*-step ahead mean-square forecast error (MSFE). Due to data availability, we keep h=1 so as to minimize further data losses. We divide the data into three equally spaced and contiguous periods: (i) initialization ($t\in \{0,\ldots ,T_{1}\}$), (ii) training ($t\in \{T_{1}+1,\ldots ,T_{2}\}$), and (iii) forecast evaluation ($t\in \{T_{2}+1,\ldots ,T\}$), in which $T_{1}=*\frac{T}{3}$ and $T_{2}=*\frac{2T}{3}$. We also use a rolling validation process as follows: We first fit the model using all data up to time $T_{1}$ and forecast ${\hat{x}}_{T_{1}+1}^{\left(\lambda_{c},\alpha_{c}\right)}$, in which $\lambda_{c}$ and $\alpha_{c}$ are fixed candidate penalty terms. We then sequentially add one observation at a time and repeat this process until $T_{2}-1$. Then, we choose the penalty terms λ and α that minimize the one-step-ahead MSFE given by

(11) $$\begin{array}{c} MSFE\left(\lambda ,\alpha \right)=\frac{1}{T_{2}-T_{1}}\sum_{t=T_{1}}^{T_{2}-1}{{\hat{x}}_{t+1}^{\left(\lambda ,\alpha \right)}-{\hat{x}}_{t+1}}_{F}^{2}. \end{array}$$

Finally, we estimate the one-step-ahead forecast accuracy using data points in $t\in \{T_{2},\ldots ,T\}$, which have not been used in the model selection procedure. To better assess the potentiality of the network in amplifying contagion across different municipalities, we remove the self-loops in the estimated network, which correspond to the influence of the local infectious population on its own future value.

#### 3.2.2. Estimating Transmission Rate in Early-Time Epidemics Networks

In this section, we assume the network structure G=〈V,E〉 as given; i.e., the set of vertices and links are already established in accordance with the network construction described in Section 3.2.1. We start from the results of the early-time dynamic of SIR models in networks described in Section 2.2. There, we show that the growth rate at early time is determined by $\lambda_{\max}\beta -\gamma$ (see Equation (8)). Therefore, the graph spectrum $\lambda_{\max}$ modulates the transmission rate parameter by either amplifying or dampening the contagion speed.

If $\lambda_{\max}\beta >\gamma$, then Equation (8) grows exponentially, while it decays when $\lambda_{\max}\beta <\gamma$. Therefore, the reproduction number (critical point) is $R_{0}=\frac{\lambda_{\max}\beta }{\gamma }$. Recall that the reproduction number in the SIR model without network is $R_{0}=\frac{\beta }{\gamma }$ [12]. Therefore, the reproduction numbers of both models differ by the graph spectrum of *A*, $\lambda_{\max}$.

Equation (8) assumes that every city in the model has single growth rate dynamics dictated by the term $\lambda_{\max}\beta -\gamma$. Changes in the epidemics spreading for each city would then be fully determined by their eigenvector centralities because growth rates are identical across cities (see Equation (8)). However, studies show that the transmission rate parameter β is dependent on local aspects of cities [7]. In contrast, the recovery rate parameter is much less variable across different places. As mentioned earlier in this study, WHO indicates an average recovery time of 14 days for mild cases. Therefore, we consider a different transmission rate for each city in the network $\beta_{i}$ while having fixed the recovery rate γ for all cities. We can still apply the classical framework of SIR in networks because, even though transmission rates are city specific, they tend to be normally distributed around some mean natural value. That is, large deviations are unusual. We empirically find this fact using our application to the Brazilian case. Mathematically, we rewrite (8) as follows:

(12) $$\begin{array}{c} x_{i}\left(t\right)\approx v_{i,1}e^{\left(\lambda_{1}\beta_{i}-\gamma \right)t}. \end{array}$$

We can linearize (12) by simply taking the $\log\left(.\right)$ at both sides of the equation for each city *i* in the network:

(13) $$\begin{array}{c} \log\left(x_{i}\left(t\right)\right)=\log\left(v_{1,i}\right)+\left(\lambda_{1}\beta_{i}-\gamma \right)t, \end{array}$$

∀i∈V. The LHS and RHS are always non-negative, because x(0)≥0 and is nondecreasing (early-time assumption), $e^{\left(\lambda_{1}\beta_{i}-\gamma \right)t}\geq 0$ (asymptotically speaking), and $v_{1,i}\geq 0$ [13]. We can then apply the $\log\left(.\right)$ without any restrictions. We can estimate (13) for all cities *i* at once by adding dummies for the constant and time-dependent term for each city in the model (2 dummies per each city). We end up with a set of 2n−1 dummy variables because the last one is the reference dummy. Since we have panel data with temporal dependencies (the same city appears multiple times), we use a linear panel data estimation model [35] as follows:

(14) $$\begin{array}{c} x_{i}\left(t\right)=\sum_{j\in V}\delta_{ij}\left[\alpha_{j}+\rho_{j}\cdot t\right]+\epsilon_{i}\left(t\right), \end{array}$$

∀i∈V, in which $\alpha_{i}$ and $\rho_{i}$ are the constant and time-variant dummy terms for city *i*, and $\epsilon_{i}\left(t\right)$ is the residual from the least square estimation with dummies. We cluster the errors at the city level to mitigate concerns with heteroskedasticity and serial correlation, which could bias our coefficient estimates. Equations (13) and (14) are linked by the following identities:

(15) $$\begin{array}{cc} \alpha_{i} & =\log\left(v_{1,i}\right)\Rightarrow v_{1,i}=e^{\alpha_{i}}, \end{array}$$

(16) $$\begin{array}{cc} \rho_{i} & =\lambda_{1}\beta_{i}-\gamma \;\Rightarrow \beta_{i}=\frac{\rho_{i}+\gamma }{\lambda_{\max}}. \end{array}$$

Given the recovery rate γ—which is assumed to not change over time or across cities—we can fully identify the eigenvector centrality and the local transmission rate of every city *i* using (15) and (16), respectively. We only take city-specific estimations of $v_{1,i}$ and $\beta_{i}$ that are statistically significant at the 10% level. Otherwise, we set the estimated coefficients to zero.

#### 3.2.3. Assessing the Efficiency of Health Policy Measures in Epidemics Spreading

With our framework, we can analyze the speed of the epidemics spreading through the network at early time by simply inspecting the graph spectrum $\lambda_{\max}=\lambda_{1}$ for different time horizons using the methodology described in Section 3.2.1. Since the reproduction number of the epidemics is proportional to the graph spectrum, then large graph spectra indicate a higher speed of contagion. Any changes in the graph spectrum can be attributed to a “net effect” of public policies of the government in the entire network. Since we use the share of infectious persons of each Brazilian city, then this “net effect” comprises not only federal policies but also state- and even city-level policies.

Moreover, we can estimate the human impact of these policies in terms of changes in the number of infectious persons at the peak by running the SIR model described in Section 3.2.2 for each estimated city-specific transmission rate parameter $\beta_{i}$ defined in (16) and for different values of the graph spectrum. We use a conservative approach and compare the largest observed graph spectrum with our dataset’s most recent graph spectrum. We assume that the largest graph spectrum occurs when public policies were still latent and did not affect the spread of epidemics. Most recent values of the graph spectrum are assumed to represent transmission dynamics after public policies were in, as was the case in Brazil, which adopted quarantine and recommended the use of masks in the period that we have available data.

Social isolation and the use of masks are not policies per se but rather strategies (or nonpharmacological interventions) aimed at containing the pandemic based on the health policies defined by each of the governments.

## 4. Results and Discussion

**Figure 1:**
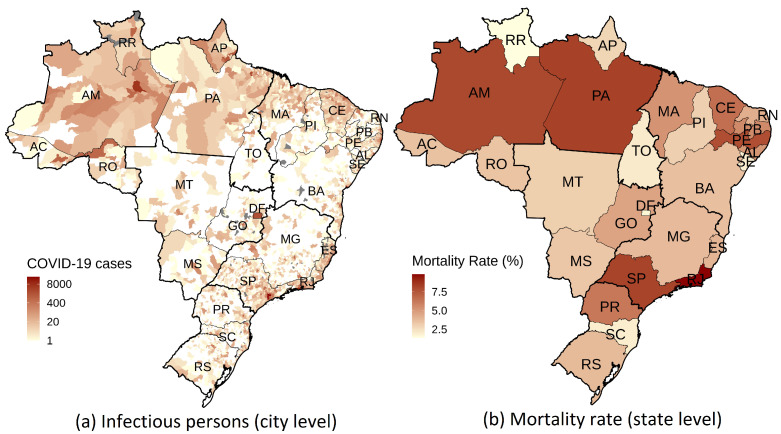
COVID-19 geographical spreading pattern in Brazil in terms of (**a**) COVID-19 cases at the city level and (**b**) mortality rate at the state level as of 8 May 2020. Gray areas represent cities that have not reported any COVID-19 epidemiological bulletin. We evaluate mortality rates by taking the ratio of the number of deaths due to COVID-19 to the number of infectious persons. Mortality rates are probably upward biased because the number of observed infectious persons is likely to be underestimated, as COVID-19 may pass un-noticed for some cases (mild or no adverse conditions at all). We report mortality rates at the state rather than city level because many cities have few COVID-19 cases and deaths, which would distort the estimated mortality rates.

This section presents the main empirical results of the paper, beginning with the construction of the COVID-19 intercity transmission network and an analysis of its propensity to amplify COVID-19 in different cities. Subsequently, we evaluate the net effectiveness of public health measures implemented by the Brazilian government. This article centers on the domestic transmission of COVID-19 in Brazil until 8 May 2020, a date by which cases had already been registered in all 27 states, as depicted in Figure 1a. Brazil has significant socioeconomic and cultural disparities across its 5570 municipalities. Therefore, COVID-19 transmission and mortality rates may largely differ across municipalities, as evidenced in Figure 1a,b. The model proposed in this paper is able to estimate these municipality-specific COVID-19 transmission rates, thus accounting for their distinctive aspects.

**Figure 2:**
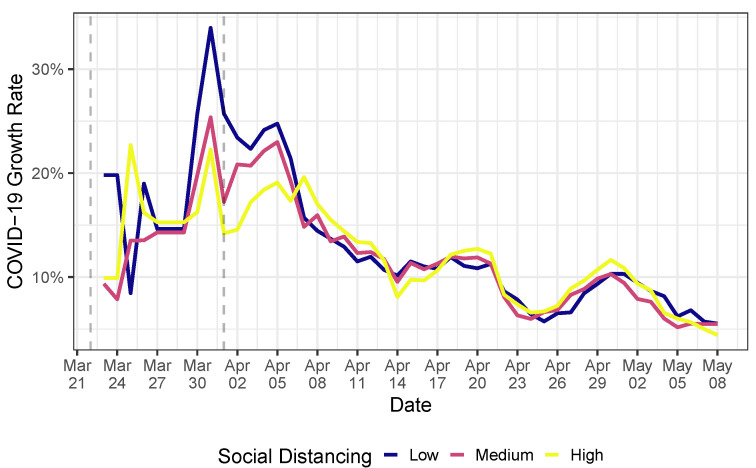
Average COVID-19 growth rate in cities of the state of São Paulo, Brazil, with low, medium, and high social distancing indices. Available data go until 8 May 2020. The first vertical line is the beginning of SP quarantine, while the second represents the use of mask recommendations by the federal government. Data from social distancing are public and come from the São Paulo State Government(https://www.saopaulo.sp.gov.br/coronavirus/isolamento/, accessed on 3 January 2021) (in Portuguese). To alleviate week seasonality, we use 7-day moving averages to construct the average growth rates. The low, medium, and high social distancing indices represent the bottom, middle, and upper terciles of the corresponding distribution. Data from the number of infectious persons per city are discussed in Section 3.1.

We find that the quarantine and use of mask measures decreased the growth rate of the spectrum of the COVID-19 transmission network over time, suggesting that the measures were effective. To illustrate, Figure 2 portrays the average COVID-19 growth rate of cities in the state of São Paulo segregated in terms of their average social distancing index in the period. (Such index represents the extent of compliance of the population to the quarantine measures.) First, after the use of mask recommendation, the COVID-19 growth rate, in general, decreased. However, it decreased more in cities of São Paulo with low social distancing measures. This may be due to the fact that these cities could have more potential close human-to-human contact, and therefore, the use of masks is crucial to detain COVID-19 transmission. To get a sense of the human impact of such measures, we build counterfactual scenarios in which we consider that none of these measures were taken by the government. By running the SIR model in networks, we find that the quarantine and the use of mask recommendation reduced the peak of the COVID-19 epidemics, on average, by 15% in São Paulo (SP) and almost 25% in Brasília (DF), when we look at the average effect in the last week of available data (2–8 May 2020). This reduction is explained by the flattening of the epidemics curve: São Paulo (SP) and Brasília (DF) have peak date shifts from 7–24 July and 29 August to 28 September, respectively.

**Figure 3:**
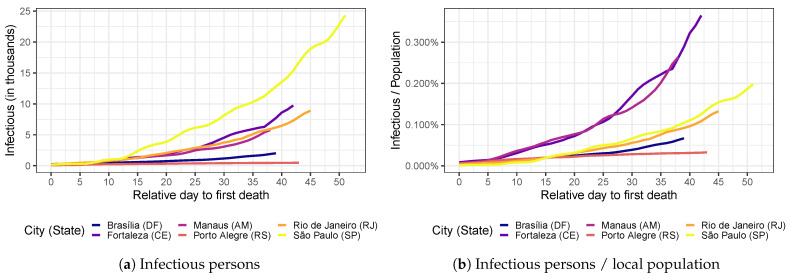
COVID-19 evolution in six of the most affected cities in Brazil (**a**) in absolute terms (number of infectious persons) and (**b**) as a share of the local population size. The horizontal axis represents the relative day in terms of the first observed death due to COVID-19.

Figure 3a,b portray the COVID-19 evolution in six of the most affected cities in Brazil relative to the first reported death in terms of the number of COVID-19 cases and as a share of the local population size, respectively. São Paulo (SP) has the highest number of infectious persons. However, there is a strong size effect: São Paulo (SP) has almost 12.2 million residents, while the second largest city, Rio de Janeiro (RJ), has almost half of that (6.8 million). To get a sense of the local COVID-19 criticality, we can look at its evolution as a share of the local population. In this case, we note that COVID-19 transmission speed is much larger in Manaus (AM) and Fortaleza (CE). Brasília (DF) and Porto Alegre (RS) have smaller transmission rates and local COVID-19 criticality. However, mortality rates may not follow such incidence criticality because they correlate with local health quality and demographic characteristics.

### 4.1. Intercity COVID-19 Transmission Network in Brazil

**Figure 4:**
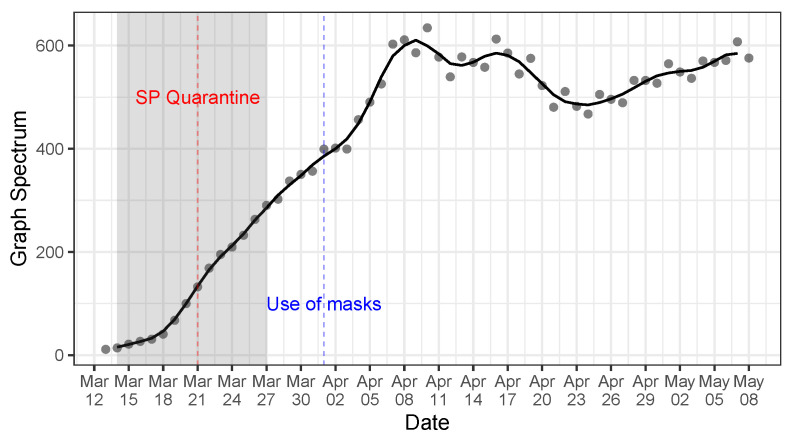
Graph spectrum of the COVID-19 intercity transmission network of Brazil (see Section 3.2.1). The shaded area indicates the timing window in which quarantine measures were adopted by the most affected Brazilian states. The red dashed line indicates the beginning of the quarantine in São Paulo, the COVID-19 epicenter in Brazil. The blue dashed line indicates the beginning of the use of masks recommended by the Federal Health Ministry. For each time point (horizontal axis), we build the network with city-specific shares of infectious persons with data up to that point.

Figure 4 shows the graph spectrum of the COVID-19 intercity transmission network of Brazil over time. For each time point (horizontal axis), we run the network construction through the fitting process in Section 3.2.1 with data from the beginning of the sample up to that specific time point. Even though our sample starts on 25 February 2020, we start the fitting process on 13 March 2020, so as to have enough data for the fitting process. That is, we start with 18 time points for each Brazilian city. Therefore, we initially divided the panel data into three equally sized groups with 6 time points for model training, model selection (parameters and penalty terms), and model evaluation. These group sizes increase as we add more time points. We perform the network construction estimation daily from 13 March to 8 May 2020 in an independent manner.

In Figure 4, we add a shaded area indicating the timing window in which quarantine measures were adopted by the most affected Brazilian states. Since São Paulo is the COVID-19 epicenter in Brazil, as it encloses 57.4% of all the COVID-19 infections, we also add a vertical dashed red line indicating the beginning of the quarantine adopted by the São Paulo State Government. We also draw the use of mask recommendation beginning date by the Federal Health Ministry in Brazil as a dashed blue line enacted. While quarantine measures are at the state level, the use of mask recommendations goes at the federal level and encompasses all the 5570 cities and 27 states in Brazil. São Paulo is the most central city in the transmission network. Therefore, it practically shapes the graph spectrum of the intercity transmission network.

Google’s PageRank (PR) is a well-known metric for centrality used to rank websites ([36]). Its purpose is to mimic the actions of an actual web surfer. By default, a user gets around the web by clicking links on their viewing page. This is also called “surfing”. To quickly navigate to a different page, you can also use the browser’s address bar to enter the page’s URL, use bookmarks, and so forth. This process can be shown on a network by combining a random walk with jumps to any nodes. One can describe this using a small set of implicit relations:

$$p\left(i\right)=\frac{q}{V}+\left(1-q\right)\sum_{j\in V:j\to i}\frac{p(j)}{k_{j}^{\left(\mathrm{out}\right)\;}}$$

*V* stands for the graph number of vertices, p(i) is the PR value of vertex *i*, and $k_{j}^{\left(\mathrm{out}\right)\;}$ is the out-degree of vertex *j*, whereas the sum occurs over the vertices pointing toward (direct connection to) *i*. The damping factor q∈[0,1] is a probability distribution function that quantifies the relative contribution of the realized random walk and random jumps.

A significant PR needs to have many neighbors pointing at it or a large in-degree. However, it is also necessary for the neighbors themselves to have large PR values. Consequently, if two vertices have the same degree, the vertex with more “important” neighbors will have a more outstanding PR.

We observe a reduction in the growth rate of the graph spectrum after the quarantine measures precisely 2 days after the measure. However, the growth rate still persisted at positive rates, indicating that the COVID-19 transmission speed kept increasing after such a measure, but at a slower pace. After the incubation period following the use of mask recommendation, we observed a drastic change in the graph spectrum. The growth rate changed sign and started to reduce, showing that the government’s set of health policy measures was efficient. However, after 23 April 2020, the graph spectrum again started to increase. This can be due to several factors, such as social confusion in following health guidelines in view of the political disarray that Brazil is facing or even noncompliance with quarantine and the use of mask measures. Our model does not permit an isolated causal impact of the use of mask recommendation or the quarantine measures. However, it enables us to understand how the set of all policy measures affected the COVID-19 transmission rate across cities over time. Combining Figure 2 and Figure 4, it seems that the reduction in the COVID-19 growth rates after the use of mask recommendation was more apparent in cities with relatively low social distancing indices. This may be due to the fact that these cities have more potential close human-to-human contact, and therefore, the use of masks is crucial to detain COVID-19 transmission.

**Figure 5:**
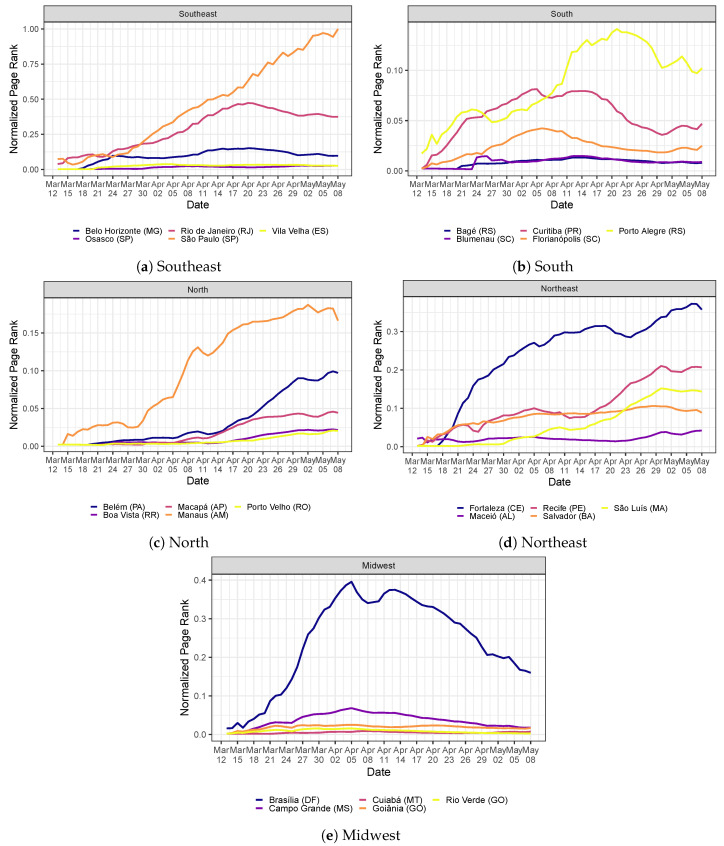
Evolution of the normalized PageRank centrality measure in the COVID-19 transmission network (see Section 3.2.1 for the network construction details). We only report the top 5 cities with the highest PageRank in each Brazilian region. For each time point (horizontal axis), we build the network with city-specific shares of infectious persons with temporal data up to that point. Each label is composed of the city name followed by its state inside parentheses.

To understand the topological aspects of the COVID-19 intercity transmission network, Figure 5 plots the PageRank centrality for the top 5 most central cities in each of the five regions in Brazil. We normalize the PageRank with respect to the most central city, São Paulo (SP), on 8 May 2020. As the city’s centrality increases, it contributes to spreading COVID-19 throughout the network. The top 5 most central cities in the country are the following state capitals (in decreasing order): (i) São Paulo (SP), (ii) Rio de Janeiro (RJ), (iii) Fortaleza (CE), (iv) Recife (PE), and (v) Manaus (AM). These cities all have airports and are strongly interconnected to the remainder of cities in Brazil through roadways and are likely to be the hubs for the COVID-19 spread to other nearby cities in Brazil, especially countryside municipalities. The centrality of São Paulo (SP) in the southeast monotonically increases over the entire sample. The same roughly occurs with Manaus (AM) in the north and Fortaleza (CE) in the northeast. Porto Alegre (RS) in the south and Brasília (DF) in the midwest have the highest centralities in their region but with a negative growth rate in the last days of the sample. Overall, there is a very heterogeneous profile of the city centralities over time, showing the underlying nontrivial patterns in the COVID-19 transmission network.

### 4.2. Measuring the Human Impact of Health Policy Measures to Mitigate the COVID-19 Propagation

In this section, we run the SIR in networks (see Equations (1)–(4)) with different transmission rate parameters for each city in Brazil, in accordance with (16). We first estimate the city-specific $\rho_{i}$ using the panel data information on counts of the share of infectious persons in each city in Brazil via (14). Then, we estimate the transmission rate parameter $\beta_{i}$ of each city i∈V in Brazil by fixing the recovery rate parameter as γ=1/14. We use the remaining parameter $\lambda_{\max}$—the graph spectrum—to evaluate the effectiveness of the set of health policy measures in detaining COVID-19 in Brazil. We take as a baseline model the graph spectrum reached on 10 April 2020, which is the maximum observed value. We assume that this graph spectrum would not have changed afterward in case the set of health policy measures was not taken. (This is a conservative approach because we can observe a positive momentum of the graph spectrum growth rate prior to reaching 10 April 2020. However, we cannot be sure whether such a graph spectrum would still increase if these policies were not in place. Therefore, we keep the conservative approach and consider such points as the maximum.) We then run several SIR models with the observed graph spectrum values in Figure 4 after the graph spectrum maximum on 10 April 2020.

**Figure 6:**
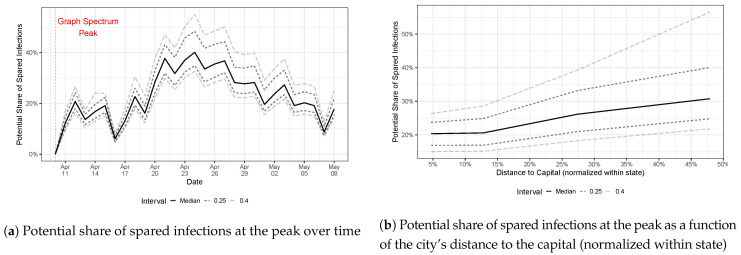
Distribution of the efficiency of health policy measures along all affected cities in Brazil over time. We plot the efficiency distribution as a function of (**a**) time and (**b**) the city’s distance to the capital within the same state it resides. Since states in Brazil have substantial differences in their sizes, we normalize the city’s distance from the capital to the most distant city within the state.

Figure 6a shows a comparison of the infectious peaks of the baseline SIR model—i.e., the hypothetical scenario in which health policy measures were not introduced—and the ones with graph spectrum values observed daily after that maximum. The vertical axis shows the relative change in these infectious peaks of the baseline and the observed model day by day, which can be interpreted in terms of the potential share of spared infections at the infectious peak due to the introduction of the set of health policy measures. Since we have data from each city affected by COVID-19, we plot the median and percentiles 75% (0.25 distant from the median) and 90% (0.40) of this distribution. On 10 April 2020, the share of spared infections in the epidemic’s peak was zero because the baseline model is compared with itself. Then, as we move forward in time and use smaller graph spectrum values, as shown in Figure 4, the potential share of spared infectious persons increases. The share of spared infectious persons in the epidemic’s peak reaches a median value 40% lower than that of the baseline model when we use the graph spectrum on 24 April 2020, suggesting the high effectiveness of the quarantine and use of mask health policies. After this point, the share of spared persons decreases—reflecting the increase in the graph spectrum in Figure 4—giving more room for the spread of COVID-19. The effectiveness of the health policy measures, however, remains positive throughout the entire sample.

The first case of COVID-19 in Brazil was reported in São Paulo (SP) on 25 February 2020. After that, it spread to several Brazilian state capitals, probably through air transportation (most of the airports in Brazil are in state capitals, and the capitals are far from each other). The epidemics took some time before reaching the first case in countryside cities. Figure 6b displays the distribution of the potential share of spared infections in terms of the city distance to the state capital. Since Brazilian states are very different in size, we normalize the distance to the most distant city within the state.

We observe a positive relationship between the potential share of spared infectious persons and distance to the capital, suggesting that public health policies are most effective in distant cities. This may reflect not only the temporal delay of COVID-19 in reaching the countryside, which puts the local COVID-19 at a very early time in these regions, but also demography aspects, such as lower population density and agricultural economic activities that do not require large conglomerates of persons.

We analyzed the effectiveness of public health measures—such as social isolation/quarantine and the use of masks—in mitigating the transmission of COVID-19 in the country, using a network-based approach. In a study conducted on the efficacy of individual and multiple public health measures in reducing the incidence of COVID-19, the transmission of SARS-CoV-2, and mortality from COVID-19, it was found that practices such as handwashing, mask wearing, and physical distancing were effective in reducing the incidence of the disease. These measures are known as nonpharmaceutical interventions [2].

Similarly, corroborating our results, another study found that nonpharmaceutical interventions played a significant role in the reduction of COVID-19. Through a systematic review, the authors evaluated the efficacy of public health interventions and concluded that such nonpharmaceutical interventions and control measures were essential [30]. Among the effective measures are not only the isolation and quarantine of confirmed cases but also social distancing, mandatory mask wearing, travel restrictions, quarantine for travelers from affected areas, urban lockdowns, mass gathering restrictions, contact tracing and testing, school closures, and the use of personal protective equipment by healthcare professionals.

In estimating the effects of these interventions in 41 countries during the first wave, the authors discovered that some measures were robustly more effective than others. Ge et al. [3] evaluated, in addition to nonpharmaceutical interventions (NPIs), the impact of vaccination programs. Upon analyzing 31 member states of the European region of the World Health Organization, they identified that NPIs significantly contributed to reducing the disease and were complementary to vaccination. The effects of both strategies were highly correlated. Vaccination only outperformed NPIs from August 2021 onward. It should be emphasized, as [32] Haug noted, that an appropriate combination of NPIs was vital to contain the virus. It was essential to prioritize efficient measures to contain the spread of the disease. These approaches take into account the intra- and intermunicipal transmission channels of COVID-19.

Like the majority of countries in the Americas, Brazil adhered to the measures imposed during the early stages of COVID-19. Eastern countries, such as China, South Korea, and Singapore, have indicated that having pre-established policies is fundamental to successfully mitigating the death toll. The efficacy of these measures varies, and some of them have a more significant impact on both the economy and the health of populations.

The development of government policies aimed at mitigating the spread of COVID-19 should consider local particularities. In the event that a government omission occurs at the onset of the epidemic, it can have significant long-term human and economic effects. In this study, we only considered the availability of initial data on COVID-19 dynamics, thus better reflecting the real-world conditions that most governments faced.

**Figure 7:**
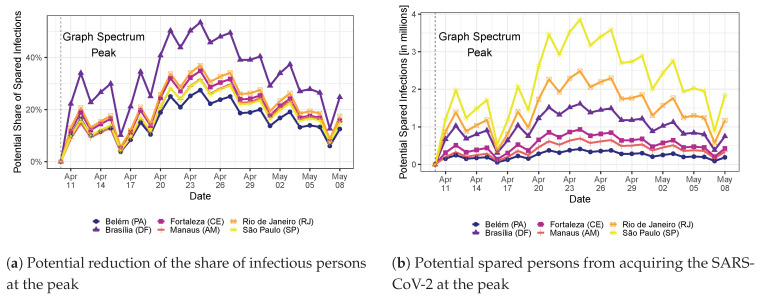
Efficiency of public health measures over time as a function of (**a**) the share of the spared local population and (**b**) the spared number of persons (in millions). We depict curves only for six capital cities that are being substantially affected by COVID-19: Belém (PA), Fortaleza (CE), Rio de Janeiro (RJ), Brasília (DF), Manaus (AM), and São Paulo (SP).

Figure 7a shows the effectiveness of the set of public health measures for six of the most affected Brazilian capitals. In particular, Brasília (DF) reaches a 50% lower share of infectious people at the peak when we compare the peaks reached with the graph spectrum value on 24 April 2020 (against the baseline on 10 April 2020). Figure 7b shows the number of potentially spared infectious persons due to the set of health policy measures. This figure is constructed simply by multiplying the share of spared infectious persons with the local population size of each of the six cities. Since São Paulo (SP) is the largest city, it would potentially spare more persons when the COVID-19 epidemic reaches its peak.

## 5. Conclusions

In this work, we present a general epidemics transmission model and apply it to the Brazilian case. One advantage of our model is that it only requires early-time data to infer the entire COVID-19 dynamics. Our method has three steps. First, we construct the COVID-19 transmission network by fitting city-specific COVID-19 cases over time to calibrate the network links, which represent intercity COVID-19 transmission. Second, we use spectral graph analysis to gauge the network propensity to spread COVID-19 throughout cities. Third, we propose a methodology to quantify the effectiveness of public health policies using the dynamics of the early-time SIR model and the spectral network theory.

Our spectral network analysis indicates that social isolation and mask use can effectively reduce the transmission rate of COVID-19 in Brazil. The dynamics of COVID-19 propagation appear to decrease after these public health policies when we also consider an incubation period, which delays the effect of any COVID-19 mitigation measure. Although we cannot isolate the effect of each policy, the use of masks seems to play an essential role in controlling epidemics, especially as countries move to a reopening of their economies [37]. With the still-limited dissemination of COVID-19 vaccines, public health intervention remains the main method of epidemic control. We hope that our study can help the government make the right decisions.

It is important to note that our analysis is not causal. We studied the associations between the relevant variables and the spread of COVID-19. Studying this link is essential in the early stages of a pandemic, for example, if people bring the virus from abroad and spread it around the municipality. As a result, we cannot attribute causality to our results, but only associations.

In Western Europe, it is clear that social isolation, although it could have prevented a rise in deaths from COVID-19, was responsible for a rise in mortality for other reasons. Children suffered from lack of social contact during their early years, many older people suffered simply from being isolated, and others went without treatment for cancer and canceled operations. The consequences of all of these issues are now becoming apparent.

Sweden managed well without government intervention. The view is that, in the future, the government’s compulsion to isolate socially will have little public support. Vaccines have been shown, without a doubt, to be the best way forward. Although there are limited data on Brazil to evaluate these aspects, it is clear that vaccination is the primary solution to the COVID-19 pandemic. Governments must exert significant pressure to achieve a more comprehensive and rapid coverage of vaccines.

It is important to note that the COVID-19 pandemic has had an effect on many financial and economic conditions in a large number of countries. Ref. [38] show the impact of COVID-19 on the international transmission of shocks across several banking systems. It also changed banks’ credit decisions ([39]). These are just some examples of the great impact that the health crisis has generated in countries and economic sectors. The measurement of these effects and the interaction between health crises and economic and financial impacts is an important avenue for further research.

## Data Availability

The data are available upon request from the authors.
